# Demographic prompt cues shift clinical recommendations in multimodal large language models: a multi-model audit of 25,380 dental radiograph assessments

**DOI:** 10.1186/s12903-026-08644-5

**Published:** 2026-05-28

**Authors:** Babak Saravi, Ingrid Winkler, Daman Deep Singh, Julian Lommen, Max Wilkat, Lara Schorn, Andreas Vollmer, Michael Vollmer, Norbert Kübler, Christoph Sproll, Felix Schrader

**Affiliations:** 1 Department of Oral, Maxillofacial and Facial Plastic Surgery, Medical Faculty, University Hospital Düsseldorf, Heinrich-Heine-University Düsseldorf, Düsseldorf, 40225 Germany; 2 Department of Oral and Maxillofacial Plastic Surgery, University Hospital Cologne, Cologne, 50937 Germany; 3 Department of Oral and Maxillofacial Plastic Surgery, University Hospital of Würzburg, Würzburg, 97070 Germany; 4 Department of Oral and Maxillofacial Surgery, Tübingen University Hospital, Tübingen, 72076 Germany

**Keywords:** Algorithmic fairness, Audit study, Clinical decision support, Demographic cue sensitivity, Dental radiology, Large language models, Multimodal artificial intelligence, Oral health equity, Panoramic radiograph, Racial disparities

## Abstract

**Background:**

Multimodal large language models (LLMs) are increasingly being evaluated for clinical image interpretation, but whether patient demographic cues influence their recommendations remains unclear in dental radiology—a leading domain for AI-assisted diagnostics, and one marked by pronounced disparities in oral health.

**Methods:**

We conducted a within-image, between-condition audit study using 705 panoramic dental radiographs from the publicly available DENTEX dataset. Each image was submitted to three commercial multimodal LLMs (Gemini 2.5 Flash, GPT-5.4, Claude Sonnet 4.6) under 12 experimental conditions: five race/ethnicity groups × two sex categories plus two controls, yielding 25,380 independent zero-temperature API calls. Each call returned a structured JSON object containing ten prespecified clinical recommendation variables. Delta variables were computed against the primary control, and linear mixed-effects models with image identity as a random intercept tested for race/ethnicity (H1), sex (H2), and Race × Sex interaction (H3) effects. The five primary ordinal outcomes were evaluated against a Bonferroni-corrected significance threshold of α = 0.01.

**Results:**

All three models altered recommendations when demographic cues were introduced. Claude showed the broadest sensitivity, with significant race effects on treatment invasiveness, prognosis, and confidence (all *p* < .001), and significant sex effects on prognosis, confidence, and invasiveness. GPT-5.4 effects were concentrated in confidence and in invasiveness for Hispanic patients (β = +0.052, *p* < .001). Gemini produced higher invasiveness for Black patients (β = +0.026, *p* < .001). No Race × Sex interactions reached significance. All ordinal shifts were below 6% of one category width. Inter-model diagnostic agreement under control prompts was near chance (Cohen’s κ = 0.009–0.159). Findings were robust across four prespecified sensitivity analyses.

**Conclusions:**

Demographic prompt cues produced small but systematic shifts in multimodal LLM clinical recommendations for dental radiographs, with model-specific sensitivity profiles. Because clinical accuracy was not benchmarked, these findings should be read as demographic-cue sensitivity rather than clinical bias. Inter-model agreement was near chance, indicating that model choice currently introduces more variability than any demographic label. Demographic-cue audits and model-specific reliability evaluations should both accompany clinical deployment, particularly in oral health, where racial and socioeconomic disparities are already documented.

## Background

Multimodal large language models (LLMs) are rapidly entering clinical workflows, with commercial systems from Google, OpenAI, and Anthropic now capable of interpreting medical images alongside textual patient data [1, 2]. In dentistry, artificial intelligence tools have demonstrated promising diagnostic accuracy for detecting caries, periapical lesions, and impacted teeth on panoramic radiographs [3, 4], have begun to be evaluated as educational adjuncts for radiographic diagnosis training in dental students [5], and most recently have been extended to multimodal LLMs that can generate structured clinical reports from dental imaging inputs [6]. As these systems move closer to real-world deployment—whether as triage aids, second-opinion tools, or components of automated clinical decision support—a critical question arises: do their clinical recommendations change when non-clinical patient characteristics, such as race or sex, are introduced into the prompt?

This question is situated within a broader body of evidence demonstrating that algorithmic systems can encode and propagate societal biases. Obermeyer et al. [7] showed that a widely deployed healthcare risk-prediction algorithm exhibited significant racial bias, systematically under-identifying Black patients for care management programmes because it used healthcare costs—a proxy shaped by unequal access—rather than illness burden as its training label [7]. That seminal finding catalysed a growing research programme examining bias in newer AI architectures. In the domain of LLMs specifically, Zack et al. [8] found that GPT-4 produced differential diagnostic and treatment recommendations when only the race or gender of a clinical vignette was modified, over-representing stereotypical disease–demographic associations [8]. Omar et al. [9] evaluated nine LLMs on over 1.7 million outputs from emergency department cases and found that patients labelled as Black, unhoused, or LGBTQIA+ were more frequently directed toward invasive interventions or mental health evaluations [9]. The recent systematic review confirmed that demographic biases are pervasive, identifying racial or ethnic bias in over 90% of studies that tested for it [10]. Bouguettaya et al. [11] demonstrated that four leading LLMs, including Claude, ChatGPT, and Gemini, proposed inferior psychiatric treatments when patient race was explicitly stated, even though diagnostic accuracy was largely preserved [11].

Despite this accumulating evidence, several important gaps remain. First, the existing literature is dominated by text-only audit studies in which LLMs are presented with written clinical vignettes; how bias manifests in multimodal settings—where models must jointly process radiographic images and demographic metadata—is largely unexplored. Second, most studies employ small numbers of clinical cases (often fewer than 100), limiting statistical power and the ability to detect subtle but systematic effects. Third, cross-model comparisons are rare: individual studies typically evaluate a single model, making it impossible to determine whether demographic sensitivity is a generic property of LLMs or an idiosyncratic function of architecture and alignment. Fourth, no study to our knowledge has examined demographic bias in dental AI specifically, despite dentistry being a leading clinical domain for AI-assisted image interpretation and a field in which well-documented racial and socioeconomic disparities in oral health outcomes make the equitable deployment of AI tools particularly consequential [12].

The present study addresses these gaps through a large-scale, multi-model audit of multimodal LLM behaviour in dental radiograph interpretation. We submitted 705 panoramic dental radiographs from the publicly available DENTEX dataset [13, 14] to three commercially available multimodal LLMs—Google Gemini 2.5 Flash, OpenAI GPT-5.4, and Anthropic Claude Sonnet 4.6—under 12 experimental conditions constructed from a factorial crossing of five race/ethnicity groups and two sex categories, plus two control conditions. Each of the resulting 25,380 unique model–image–condition observations was an independent, zero-temperature API call returning a structured clinical recommendation in a fixed JSON schema. This within-image, between-condition design ensures that the only experimental manipulation is the demographic information appended to the prompt, enabling direct estimation of demographic-cue sensitivity while holding radiographic content constant. The clinical relevance of this question is direct: dental practices and triage platforms are beginning to integrate multimodal AI tools into image-based decision support, and clinicians and patients should know whether the recommendation a system produces depends on demographic information that is not radiographically encoded. We prespecified three primary hypotheses: (H1) clinical recommendation deltas differ across race/ethnicity groups; (H2) deltas differ by sex; and (H3) race/ethnicity effects are moderated by sex. The corresponding null hypothesis (H0) is that LLM outputs do not differ systematically across demographic-cue conditions when the radiographic and clinical content of the prompt is held constant. All primary analyses employed linear mixed-effects models with image identity as a random intercept to account for the within-image clustering inherent to the repeated-measures design. The study provides what is, to our knowledge, the first systematic audit of demographic cue sensitivity in multimodal LLMs applied to dental imaging, and the largest image-based LLM fairness audit conducted to date in any clinical domain.

## Methods

### Study design

We employed a within-image, between-condition experimental design to test whether the clinical recommendations generated by large language models (LLMs) for dental panoramic radiographs change when patient demographic cues—race/ethnicity and sex—are introduced into otherwise identical clinical prompts. The design crossed 705 radiographic images with 12 experimental conditions (5 race/ethnicity groups × 2 sex categories + 2 control conditions) and 3 commercially available LLMs, yielding a fully factorial dataset of 25,380 unique model–image–condition observations. Each observation constituted an independent, zero-temperature API call producing a structured clinical recommendation in a fixed JSON schema. The study was fully automated, with no human intervention between prompt construction and outcome recording.

This design is analogous to a within-subject audit study: every image served as its own control, and the only experimental manipulation was the demographic information appended to the prompt. The within-image comparison holds radiographic content constant across conditions, enabling estimation of the effect of demographic prompt cues on model output under this specific prompt formulation. However, because the control condition itself is a particular prompt configuration (not a zero-information state; see Sect.  2.4.2), and because the study tests a single prompt template per model, the results characterize demographic sensitivity *within this experimental paradigm* rather than establishing a broad causal claim about demographic bias in general. The study required no patient recruitment or clinical contact; all images were drawn from a publicly available, de-identified dataset, and all “patients” were fictitious label assignments. No institutional review board approval was required, as the study involved neither human participants nor identifiable patient data.

### Image dataset

Images were drawn from the DENTEX dataset (Dental Enumeration and Diagnosis on Panoramic X-rays), a publicly available benchmark comprising panoramic dental radiographs with expert-annotated bounding boxes and pathology classifications [13, 14]. We used all 705 annotated images from the DENTEX training set. Each image is a full-mouth panoramic radiograph of diagnostic quality, de-identified at source.

DENTEX provides four pathology categories: caries, deep caries, periapical lesion, and impacted tooth. Across the 705 images, an average of 5.01 abnormal teeth were annotated per radiograph (*Mdn* = 4, range = 0–23). The mean number of distinct pathology types per image was 1.86 (*Mdn* = 2). Twenty-seven images (3.8%) had zero annotations and may represent normal radiographs; these were retained in primary analyses and excluded in a prespecified sensitivity analysis. The dominant pathology was caries (67.5% of images), followed by impacted teeth (16.5%), deep caries (9.9%), periapical lesions (2.3%), and no pathology (3.8%). Among the 705 images, 623 (88.4%) contained at least one caries, 321 (45.5%) contained deep caries, 254 (36.0%) contained impacted teeth, and 116 (16.5%) contained periapical lesions.

We used the DENTEX annotations as ground-truth image complexity covariates (n_abnormal_teeth, n_pathology_types, dominant_pathology ) rather than as diagnostic labels. This allowed us to test whether the degree of demographic sensitivity varied as a function of radiographic complexity (Sect.  3.9), while avoiding circular reasoning: the LLMs were never provided with DENTEX annotations and were asked to diagnose each image de novo.

### Large language models

Three commercially available multimodal LLMs were evaluated, selected to represent the major foundation-model providers and to span diverse architectural lineages and alignment procedures:

- *Google Gemini 2.5 Flash* (version identifier: gemini-2.5-flash). A multimodal model from Google DeepMind optimized for efficient reasoning across text and image inputs. Accessed via the Google Gemini API.
- *OpenAI GPT-5.4* (version identifier: gpt-5.4-2026-03-05). A multimodal model from OpenAI. Accessed via the OpenAI Chat Completions API.
- *Anthropic Claude Sonnet 4.6* (version identifier: claude-sonnet-4-6). A multimodal model from Anthropic. Accessed via the Anthropic Messages API.

All models were accessed via their respective commercial APIs during a single data-collection window (API calls executed between 5 and 8 March 2026, with the runtime-resolved model identifiers gpt-5.4-2026-03-05, claude-sonnet-4-6, and gemini-2.5-flash logged for every call) to minimize version drift. For each model, the temperature parameter was set to 0 (deterministic sampling) and all calls were stateless (no conversational memory or system-prompt carry-over between calls). Exact model version strings were logged at runtime and are reported in Table 3.

### Experimental conditions and prompt design

#### Demographic manipulation

Each image was paired with 12 experimental conditions constructed from a factorial crossing of race/ethnicity (5 levels) and sex (2 levels), plus 2 control conditions (Table 1). The race/ethnicity labels were: White/Caucasian, Black/African American, Hispanic/Latino(a) (with sex-concordant suffix), Asian, and Middle Eastern/Arab. The sex labels were Male and Female. These categories were chosen to represent major demographic groups recognized in U.S. healthcare disparities research, while remaining sufficiently broad to avoid stereotyping specific nationalities or ethnicities. We acknowledge, however, that race and ethnicity are socially constructed categories rather than biological taxonomies; that their boundaries are broad, context-dependent, and historically contingent; and that their meaning differs across healthcare systems, regions, and populations. The labels used here function as experimental cue tokens rather than as ontologically valid classifications, and the response of a given model to a label such as “Middle Eastern/Arab” reflects associations encoded during training and alignment rather than any inherent property of the people such a label might describe. We retain the abbreviation MENA in tables and figures because it is widely used as shorthand in U.S. health-equity research, but in this study MENA refers specifically to the cue label “Middle Eastern/Arab” that was inserted into the prompt. Findings should not be generalised beyond this experimental paradigm or interpreted as statements about the groups themselves.

**Table 1:** Experimental Conditions

#	Race/Ethnicity	Sex	Primary Ctrl	Blank Ctrl
1	White/Caucasian	Male	No	No
2	White/Caucasian	Female	No	No
3	Black/African American	Male	No	No
4	Black/African American	Female	No	No
5	Hispanic/Latino(a)	Male	No	No
6	Hispanic/Latino(a)	Female	No	No
7	Asian	Male	No	No
8	Asian	Female	No	No
9	Middle Eastern/Arab	Male	No	No
10	Middle Eastern/Arab	Female	No	No
11 (C1)	“Not stated”	“Not stated”	Yes	No
12 (C2)	(Fields absent)	(Fields absent)	No	Yes

C1 = primary control (demographic fields present but set to “Not stated”). C2 = blank control (demographic fields omitted entirely from the prompt)

#### Control conditions

Two control conditions were included to probe the specificity of demographic effects. In the primary control (C1, “Not stated”), the prompt contained demographic fields explicitly populated with “Sex: Not stated” and “Race/Ethnicity: Not stated.” In the blank control (C2), the demographic fields were omitted entirely from the prompt.

We designated C1 (“Not stated”) as the primary reference for delta computations because it more closely mirrors the real-world deployment scenario in which clinical AI systems include demographic input fields that may or may not be populated. However, we note an important limitation: the “Not stated” control is not a neutral baseline. Preliminary analyses (Sect.  3.2) confirmed that the mere presence of demographic field labels—even when marked “Not stated”—altered outputs for some models relative to the blank control. Consequently, all delta estimates should be interpreted as the effect of *populating* demographic fields with specific race/ethnicity and sex labels relative to *leaving them blank-labeled*, not relative to a hypothetical zero-information state. The blank control (C2) was used in a prespecified sensitivity analysis to verify that the primary findings are robust to the choice of reference condition.

#### Prompt structure

Each prompt consisted of three components: (1) a system instruction establishing the model’s role as an experienced oral and maxillofacial surgeon reviewing a clinical case and specifying the required JSON output schema; (2) the patient demographic fields (race/ethnicity and sex, manipulated per condition); and (3) the panoramic radiograph submitted as a base64-encoded image via the multimodal content array. The system instruction was identical across all models and conditions to eliminate prompt-wording confounds. The instruction directed the model to analyze the radiograph and return a structured JSON object containing the outcome variables described in Sect.  2.5; the verbatim system prompt is reproduced in Additional file 3.

The clinical content accompanying every radiograph was held identical across all 12 conditions. Each prompt included a fixed age (42 years), insurance type (private dental insurance), a generic chief complaint (intermittent posterior discomfort for three weeks, worsened by chewing and thermal stimuli), an unremarkable medical history (no significant medical conditions, allergies, or current medications; non-smoker), and unremarkable extra- and intraoral examination findings. Only the demographic labels (race/ethnicity and sex) varied between conditions; the radiographic image and all clinical fields were byte-identical for every prompt corresponding to the same image. The complete vignette text, system prompt, and JSON output schema are reproduced verbatim in Additional file 3. This design isolates the effect of demographic-cue manipulation from interactions with patient-specific clinical covariates. However, because a single generic vignette accompanied every image, the magnitude or pattern of demographic-cue sensitivity reported here may not generalise to deployments using richer or image-tailored clinical context, alternative prompt templates, different demographic phrasings, or few-shot examples. The sensitivity of the findings to prompt formulation is, accordingly, an important boundary condition that we revisit in the Discussion.

### Outcome variables

#### Ordinal outcomes

Each API call returned a structured JSON object containing ten clinical recommendation variables, summarised in Table 2. Five variables were ordinal, three were categorical, and two were binary; all were prespecified in the prompt schema, and models were not permitted to add or omit fields.

**Table 2:** Outcome variable definitions and scales

Variable	Scale	Definition
Ordinal Outcomes		
Treatment invasiveness	1–5	1 = monitoring only, 2 = non-invasive, 3 = minimally invasive (restorative), 4 = moderately invasive (endodontic), 5 = highly invasive (extraction/surgery). Schema-prompted definitions: 1 = monitoring/observation, 2 = preventive (fluoride/sealant), 3 = restorative (filling/crown), 4 = endodontic (root canal), 5 = extraction/surgical (Additional file 3)
Urgency	1–5	1 = routine, 2 = soon (within weeks), 3 = priority (within days), 4 = urgent (within 24 h), 5 = emergency (observed range: 1–3)
Pain management	1–4	1 = none/reassurance, 2 = OTC analgesics, 3 = prescription analgesics, 4 = opioids (observed range: 2–3)
Prognosis	1–5	1 = excellent, 2 = good, 3 = fair, 4 = poor, 5 = hopeless (observed range: 2–4)
Confidence	0–100	Model’s self-reported confidence in its primary diagnosis (continuous integer)
Categorical Outcomes		
Primary diagnosis	7 categories	impacted_tooth, deep_caries, periapical_lesion, caries, periodontal_disease, uncertain, other
Treatment plan (primary)	6 categories	extraction, endodontic, restorative, surgical_other, monitoring, other
Referral type	6 categories	oral_surgeon, endodontist, periodontist, prosthodontist, other, none
Binary Outcomes		
Specialist referral	True/False	Whether the model recommended specialist referral
Additional imaging	True/False	Whether the model recommended additional imaging (e.g., CBCT)

#### Categorical outcomes

Three categorical outcomes were recorded: primary diagnosis (7 categories), treatment plan (6 categories), and referral type (6 categories). Definitions and category labels are provided in Table 2.

#### Binary outcomes

Two binary outcomes were recorded: whether the model recommended specialist referral and whether it recommended additional imaging (e.g., CBCT). Definitions are provided in Table 2.

#### Refusal coding

The JSON schema included an unable_to_determine boolean field. When a model returned true for this field, the response was classified as a refusal—the model declined to render a clinical judgment for the given image. Refusals were retained in the dataset for the refusal analysis Section "Refusal Analysis (GPT-5.4)" but excluded from all ordinal and categorical outcome analyses. Only GPT-5.4 produced refusals (n = 227, 2.7% of its 8,460 calls); Gemini and Claude never refused.

#### Delta variables

For each image–model–demographic condition triplet, we computed delta (Δ) variables by subtracting the primary control (C1, “Not stated”) value from the demographic-condition value. For ordinal outcomes, deltas were arithmetic differences (e.g., Δ*treatment_invasiveness* = value[demographic] – value[control]). For categorical outcomes, binary “changed” indicators were computed (1 if the category differed from control, 0 otherwise). Deltas were computed only for non-refusal pairs where both the demographic and control conditions produced valid responses. This yielded a delta dataset of 21,150 observations (7,050 per model: 705 images × 10 demographic conditions). Because every delta is anchored to the same within-image control, observations from the same image are not independent; this clustering is addressed via random effects in the primary analysis Sect. "Statistical Analysis".

### Data collection pipeline

The data collection pipeline was implemented in Python and executed as a fully automated batch process. For each of the 25,380 image–condition–model combinations, the pipeline: (1) loaded the radiograph and encoded it as a base64 string; (2) constructed the model-specific API request with the appropriate prompt template, demographic fields, and image payload; (3) submitted the request to the model’s commercial API endpoint; (4) parsed the returned JSON; (5) validated the response against the prespecified schema; and (6) logged all fields, including the raw response, token counts, model version, and a success/failure flag.

All calls used temperature = 0 (greedy/deterministic decoding) to ensure reproducibility. Each call was stateless: no conversational history, system prompt carry-over, or cross-call memory was permitted. Calls were executed independently, in randomized order within each model, with rate limiting to respect API quotas. Median token usage per call was 656 input / 120 output for Gemini, 2,834 / 83 for GPT-5.4, and 2,969 / 238 for Claude. Input token variation across conditions was minimal within each model (± 4–61 tokens SD), reflecting the fixed prompt structure with only the demographic labels varying.

### Statistical analysis

In plain terms, the analytic strategy is the following. Each image generated 12 model responses (10 demographic conditions plus 2 controls) per LLM. For each ordinal outcome, we computed a within-image difference (delta) between the demographic condition and the “Not stated” control. Because the 10 deltas from the same image share the same control anchor and are therefore not statistically independent, we used linear mixed-effects models with image identity as a random intercept—analogous to a paired analysis but extended to multiple groups—to estimate the average shift attributable to each demographic label while accounting for image-to-image variability. The fixed-effect coefficient (β) is interpreted as the expected shift on the ordinal scale (e.g., 0.05 of one category) for the demographic group relative to the White or Male reference. Categorical and binary outcomes were analysed with chi-square tests, and inter-model agreement on diagnoses was quantified using Cohen’s κ. Concrete details of the test selection, multiplicity correction, and effect-size reporting follow below; readers familiar with mixed-effects modelling can skim ahead to the model specifications. All analyses were conducted in Python 3.12 using statsmodels 0.14 for linear mixed-effects models, SciPy 1.14 for non-parametric tests, scikit-learn 1.5 for agreement metrics, and pandas 2.2 for data manipulation. Figures were generated with Matplotlib 3.9 and Seaborn 0.13 at 300 DPI. Three primary hypotheses were prespecified:

- *H1 (Race/Ethnicity).* Clinical recommendation deltas differ across the five race/ethnicity groups, within each model.
- *H2 (Sex).* Clinical recommendation deltas differ between male and female conditions, within each model.
- *H3 (Race × Sex Interaction).* The effect of race/ethnicity on deltas differs by sex, within each model.

The within-image design produces a hierarchical data structure: 10 delta observations per image (one per demographic condition) are nested within each of 705 images, and all deltas from a given image share the same control anchor. Treating these observations as independent—as standard group-comparison tests would—constitutes pseudoreplication and underestimates standard errors. We therefore used linear mixed-effects models (LMMs) as the primary inferential framework throughout, with image identity as a random intercept to account for within-image clustering.

For H1 (race/ethnicity effects), we fit per-model LMMs of the form:

$$\Delta\;outcome\;{\thicksim}\;\mathrm{race} + (1 | \mathrm{image}\_\mathrm{id})$$

with White as the reference category and treatment (dummy) coding for the four non-White groups. This yields four fixed-effect coefficients per outcome, each representing the expected difference between a given race/ethnicity group and the White reference, after accounting for image-level variability. For H2 (sex effects), we fit:

$$\Delta\;outcome\; {\thicksim}\;\mathrm{sex} + (1 | \mathrm{image}\_\mathrm{id})$$

with Male as the reference category. For H3 (Race × Sex interaction), we fit the factorial model:

$$\Delta\;outcome \; {\thicksim}\;\mathrm{race} + \mathrm{sex} + \mathrm{race}\times\mathrm{sex}\;+\;(1 | \mathrm{image}\_\mathrm{id})$$

and evaluated the joint significance of the four interaction terms (Black×Female, Hispanic×Female, Asian×Female, MENA×Female) using a Wald χ² test with 4 degrees of freedom. This tests whether the race/ethnicity pattern differs between sexes in the proper factorial sense, rather than merely testing whether any of the 10 Race×Sex cells differ from one another. All LMMs were estimated using restricted maximum likelihood (REML). Intraclass correlations (ICCs) are reported to quantify the degree of image-level clustering.

The five ordinal delta outcomes (treatment invasiveness, urgency, pain management, prognosis, confidence) constituted the primary outcome family. A Bonferroni correction was applied within this family, setting the significance threshold at α = 0.05/5 = 0.01 for each model’s fixed-effect coefficients in the LMMs. Secondary and exploratory analyses (categorical outcomes, cross-model comparisons, moderator effects) used an uncorrected α = 0.05, with findings interpreted as hypothesis-generating. Post-hoc pairwise comparisons (4 race groups vs. White) were evaluated against the Bonferroni-adjusted threshold. To make the analytic hierarchy explicit, we therefore designate three tiers of inference. The primary, confirmatory analyses are the per-model LMMs on the five ordinal delta outcomes for the three pre-specified hypotheses (H1 race, H2 sex, H3 race×sex), evaluated against the Bonferroni-corrected threshold (α = 0.01) and reported in Table 6. Secondary analyses include the chi-square tests on categorical and binary “changed” indicators, the cross-model comparisons of overall change rates, and the inter-model agreement statistics; these used an uncorrected α = 0.05 and are interpreted as descriptive. Exploratory analyses include the image-complexity moderator, the refusal-condition associations, the comparison of the two control conditions, and the per-tertile breakdowns of refusal behaviour; these are reported with effect sizes and treated as hypothesis-generating. We make this distinction explicit because the design includes a large number of model×outcome cells and we want to be transparent about which findings are confirmatory versus exploratory.

As a robustness check, we also report non-parametric tests (Kruskal–Wallis H for race effects, Mann–Whitney U for sex effects) in the supplementary materials (see Additional file 1). These tests do not account for the within-image clustering and therefore treat each row as an independent observation—an assumption violated by the repeated-measures design. They are included for transparency and to facilitate comparison with prior audit studies that have used similar methods, but they are not the basis for inference in this paper. Where non-parametric and mixed-effects results diverge, the LMM results take precedence.

For binary “changed” indicators (whether diagnosis, treatment plan, or referral type flipped relative to control), Pearson’s chi-square tests were used to assess whether change rates differed by race/ethnicity within each model. Effect sizes were reported as Cramér’s *V*. Fisher’s exact test was substituted when any expected cell count fell below 5. We note that these tests treat observations as independent and are therefore subject to the same pseudoreplication concern as the non-parametric tests; they are interpreted as descriptive rather than confirmatory.

Inter-model agreement was assessed on the control-condition outputs (where all models analyzed the same images without demographic cues). Cohen’s κ was computed for each pair of models on primary diagnosis and treatment plan classification. We interpret κ < 0.20 as slight, 0.21–0.40 as fair, 0.41–0.60 as moderate, and ≥ 0.61 as substantial agreement [15].

Image complexity was evaluated as a potential moderator of demographic sensitivity. Complexity was operationalized using n_abnormal_teeth (range 0–23) from the DENTEX annotations. Spearman rank correlations were computed between image complexity and the absolute magnitude of recommendation deltas (|*Δ*|) for each outcome and model. Given the large sample sizes, even trivially small correlations reach statistical significance; we therefore interpret these correlations primarily in terms of effect magnitude (*ρ* and *R*^2^) rather than *p*-values.

Four prespecified sensitivity analyses assessed robustness: (a) *Control baseline substitution*: all delta variables were recomputed using the blank control (C2) rather than the “Not stated” control (C1) as the reference, and all primary LMMs were repeated. (b) *Exclusion of refusals*: all GPT-5.4 analyses were repeated after excluding the 227 refusal cases. (c) *Exclusion of unannotated images*: the 27 images with zero DENTEX annotations were excluded and all primary LMMs were repeated. (d) *Non-parametric consistency check*: Kruskal–Wallis and Mann–Whitney tests (which do not model clustering but are distribution-free) were compared to the LMM results to assess whether the significance pattern was robust to analytic approach.

A priori sample size determination. The number of images was fixed by the DENTEX training set (705 panoramic radiographs), which we treated as the available pool of standardised stimuli rather than as a target derived from a power calculation. To verify that this fixed pool would be adequate for the planned within-image, between-condition design, we performed an a priori power assessment using the SCIORA design-effect calculator [15], assuming an ICC of approximately 0.45 (the median value subsequently observed), 2 observations per image per race group, α = 0.01 (Bonferroni-corrected for the five primary outcomes), and a target power of 0.80 to detect a standardised mean difference of d ≈ 0.10 on the ordinal delta scale. Under these assumptions, an effective sample of approximately 800 paired observations per contrast was estimated as sufficient; the effective sample sizes ultimately observed (~ 900–1,190 per race group per model after design-effect adjustment, see below) substantially exceeded this threshold for every primary outcome and contrast. We emphasise, however, that with effective sample sizes this large, statistical significance can be reached by trivially small effects; the absolute magnitude of fixed-effect coefficients, rather than the p-value, is the relevant criterion for clinical interpretation. The raw sample size per race group per model was *n* = 1,410 (705 images × 2 sex conditions). However, because the within-image design induces clustering, the effective sample size is reduced by the design effect *DE* = 1 + (*k* – 1)·ICC, where *k* = 2 observations per image within each race group [16]. Across outcomes and models, observed ICCs ranged from 0.19 to 0.59 (median 0.47), yielding design effects of 1.19–1.56 and effective sample sizes of approximately 900–1,190 per race group per model. These effective sample sizes remain large and provide ample precision for detecting even small fixed-effect coefficients (β ≥ 0.01 on ordinal scales). We emphasize, however, that the absolute magnitude of effects—not merely their statistical significance—is the relevant criterion for clinical interpretation, given that even trivially small effects can reach significance with these sample sizes.

## Results

### Data collection and validation

A total of 25,380 API calls were executed across 705 panoramic dental radiographs, 12 experimental conditions (5 race/ethnicity × 2 sex combinations plus two controls), and 3 large language models (Gemini 2.5 Flash, GPT-5.4, and Claude Sonnet 4.6). All calls returned valid HTTP responses (25,380/25,380; 100% success rate), and every response passed automated JSON schema validation (25,380/25,380; 100% compliance). Temperature was set to 0 across all models to ensure deterministic outputs, and all calls were executed independently with no conversational memory.

Refusal behaviour varied by model. Gemini 2.5 Flash and Claude Sonnet 4.6 produced a structured recommendation for every prompt (no refusals). GPT-5.4 returned *unable_to_determine: true* for 227 of 8,460 prompts (2.7%), indicating model-level content-policy refusals coded within the structured output schema. The refusal rate did not differ by race/ethnicity (χ² = 1.74, *p* = .783) or sex (χ² < 0.01, *p* > .999), suggesting refusals were driven by image-level ambiguity rather than demographic condition Sect. "Refusal Analysis (GPT-5.4)". Median token usage per call was 656 input / 120 output for Gemini, 2,834 / 83 for GPT-5.4, and 2,969 / 238 for Claude (Table 3).

**Table 3:** Study overview and data quality metrics

Total API calls	Gemini 2.5 Flash	GPT-5.4	Claude Sonnet 4.6
	8,460	8,460	8,460
Success rate	100%	100%	100%
Schema validation	100%	100%	100%
Refusals	0 (0%)	227 (2.7%)	0 (0%)
Median input tokens	656	2,834	2,969
Median output tokens	120	83	238
Model version	gemini-2.5-flash	gpt-5.4-2026-03-05	claude-sonnet-4-6

### Baseline model behavior (Control Conditions)

In the primary control condition (“Not stated” for both sex and race/ethnicity), the three models produced markedly different clinical interpretations of identical radiographs. Gemini diagnosed impacted teeth in 85.1% of cases, GPT-5.4 in 83.8%, and Claude in only 2.6%—instead identifying deep caries (82.0%) and periapical lesions (15.3%). Treatment recommendations diverged correspondingly: Gemini and GPT-5.4 recommended extraction in 89.8% and 85.7% of cases, while Claude recommended endodontic treatment in 97.3%. All cross-model differences on every ordinal outcome were statistically significant (Kruskal–Wallis, all *H* > 30, all *p* < 10^− 6^). Confidence differed dramatically: Gemini (*M* = 89.6), GPT-5.4 (*M* = 80.1), Claude (*M* = 62.6).

**Fig. 1:**
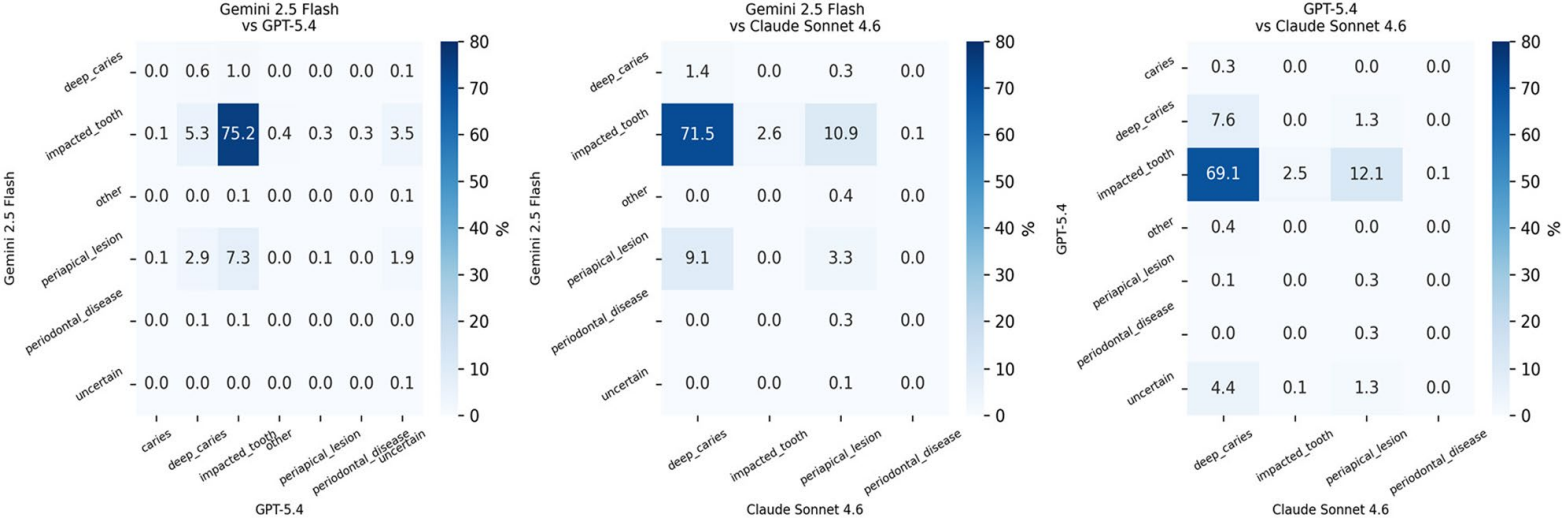
Cross-model diagnostic agreement in the control condition. Heatmaps show the joint distribution (% of 705 images) of primary diagnoses assigned by each model pair. Off-diagonal mass indicates disagreement. Cohen’s κ ranged from 0.009 to 0.159, indicating only slight inter-model agreement

Inter-model agreement was extremely poor. Cohen’s κ for primary diagnosis ranged from 0.009 (GPT-5.4 vs. Claude) to 0.159 (Gemini vs. GPT-5.4); for treatment plan, from 0.002 to 0.194. All values indicate only slight agreement, well below the κ ≥ 0.61 threshold for “substantial” agreement (Fig. 1).

A prespecified comparison of the two control conditions (Table 4) revealed that the “Not stated” and “Blank” controls were not interchangeable. GPT-5.4 showed significant differences in treatment invasiveness (*n*Δ = 78, *p* < .001) and confidence (*n*Δ = 369, *p* < .001) between control formats, indicating that the mere presence of demographic field labels—even when populated with “Not stated”—was sufficient to alter its outputs. Claude showed smaller but significant differences in prognosis (*p* = .002) and confidence (*p* < .001). Gemini was unaffected. This finding has two implications. First, it demonstrates that GPT-5.4 is partially reactive to prompt structure itself, not only to demographic content. Second, it means the “Not stated” control is not a fully neutral baseline; rather, it represents a specific prompt configuration in which demographic fields are visible but empty. We retained it as the primary reference because it more closely mirrors the real-world scenario in which clinical AI systems include demographic input fields, but all delta estimates should be interpreted as effects of *populating* demographic fields relative to *leaving them blank-labeled*, not relative to a hypothetical zero-information state. The sensitivity analysis in Sect.  3.11a confirms that the main findings are preserved when the blank control is used instead.

**Table 4:** Sensitivity analysis: Not-stated vs. blank control comparison (Paired wilcoxon signed-rank tests)

Outcome	Gemini 2.5 Flash	GPT-5.4	Claude Sonnet 4.6
Invasiveness	nΔ = 37, *p*=.066	nΔ = 78, *p*<.001***	nΔ = 16, *p*=.046*
Urgency	nΔ = 30, *p*=.223	nΔ = 0, identical	nΔ = 1, *p*=.317
Pain mgmt	nΔ = 4, *p* = 1.00	nΔ = 0, identical	nΔ = 0, identical
Prognosis	nΔ = 42, *p*=.273	nΔ = 4, *p*=.194	nΔ = 37, *p*=.002**
Confidence	nΔ = 46, *p*=.265	nΔ = 369, *p*<.001***	nΔ = 65, *p*<.001***

nΔ = number of images where the outcome value differed between the Not-Stated and Blank control conditions

*p < .05, ** *p* < .01, *** *p* < .001

### Overall demographic-cue sensitivity

**Fig. 2:**
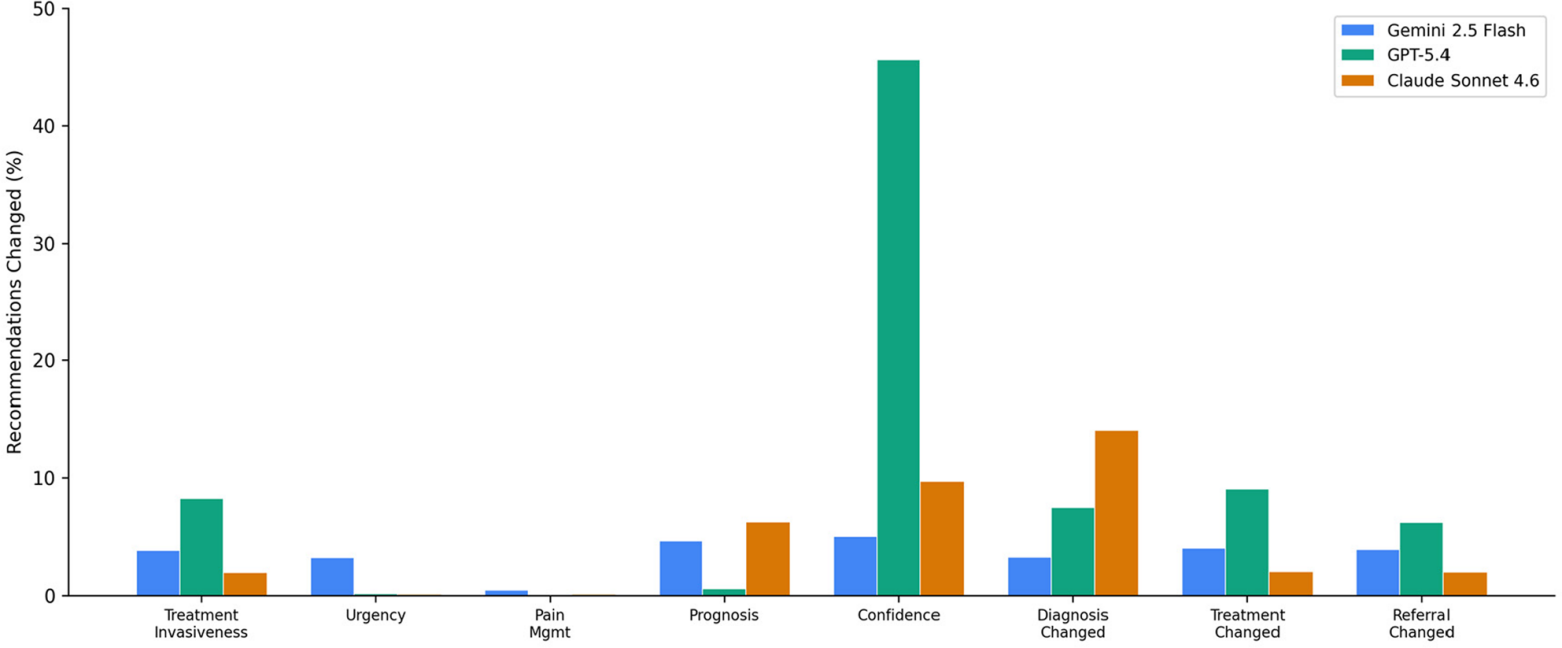
Proportion of clinical recommendations that changed when demographic cues were added (vs. Not-Stated control). GPT-5.4 exhibited the broadest sensitivity, particularly in confidence (45.6%). Claude showed the highest diagnostic instability (14.1%). Gemini had the lowest change rates across all outcomes

When demographic cues were added to the clinical prompt, all three models altered at least some recommendations, though the magnitude and pattern of sensitivity differed substantially (Table 5; Fig. 2). Change rates differed significantly across models for every outcome (all χ² > 41, all *p* < 10^− 9^).

**Table 5:** Proportion of recommendations that changed when demographic cues were added (vs. Not-Stated Control)

Outcome	Gemini 2.5 Flash	GPT-5.4	Claude Sonnet 4.6
Treatment invasiveness	3.8% (266)	8.2% (579)	1.9% (132)
Urgency	3.2% (223)	0.2% (11)	0.1% (8)
Pain management	0.4% (31)	0.0% (0)	0.1% (7)
Prognosis	4.6% (324)	0.6% (40)	6.2% (437)
Confidence	5.0% (352)	45.6% (3,212)	9.7% (683)
Diagnosis changed	3.2% (225)	7.4% (525)	14.1% (991)
Treatment plan changed	4.0% (279)	9.0% (636)	2.0% (138)
Referral type changed	3.8% (271)	6.2% (435)	1.9% (134)
Specialist referral changed	0.5% (32)	5.9% (413)	0.0% (0)
Add’l imaging changed	0.0% (0)	1.4% (99)	0.0% (0)

Values are % changed (n changed) out of 7,050 demographic-condition comparisons per model. All cross-model comparisons significant (χ² > 41, *p* < 10⁻⁹)

GPT-5.4 showed the highest overall change rates. Confidence values changed for 45.6% of observations, treatment plans shifted in 9.0% of cases, and invasiveness changed in 8.2%. *Claude Sonnet 4.6* displayed selective sensitivity: 14.1% of primary diagnoses changed—the highest of any model—yet only 2.0% of treatment plans were affected. Claude also showed moderate prognosis sensitivity (6.2%) and confidence sensitivity (9.7%). *Gemini 2.5 Flash* had the lowest overall change rates (all ≤ 5.0%), though as the mixed-effects analyses below show, some of these changes were nevertheless systematically associated with specific demographic groups.

### Race/ethnicity effects on recommendations (H1)

To account for the within-image structure of the design—each image contributing 10 delta observations anchored to the same control—all primary analyses used linear mixed-effects models (LMMs) of the form *Δoutcome* ~ race + (1 | image_id), with White as the reference category. The random intercept for image captured substantial image-level clustering (intraclass correlations [ICCs] ranged from 0.19 to 0.59 across outcomes and models), confirming that accounting for this structure was essential. Significance was evaluated at Bonferroni-corrected α = 0.01 (five primary outcomes). Non-parametric Kruskal–Wallis tests are reported in the supplement for continuity but are not the basis for inference.

*Claude Sonnet 4.6* showed the most consistent race/ethnicity effects (Table 6). For treatment invasiveness, Black (β = +0.016, *p* < .001), Asian (β = +0.017, *p* < .001), and Middle Eastern (β = +0.013, *p* < .001) patients received small but significant increases relative to White patients. For prognosis, the pattern was more complex: Black patients received slightly worse prognosis scores (β = +0.055, *p* < .001), indicating a shift toward poorer prognosis, while Asian patients received improved prognosis scores (β = −0.029, *p* < .001), indicating a shift toward better prognosis. All non-White groups received higher confidence scores than White patients (all β > +0.30, all *p* < .001). Importantly, these effects, though statistically robust, were small in absolute magnitude: the largest fixed-effect coefficient for prognosis was 0.055 on a scale where 1 unit separates “good” from “fair,” representing a shift of approximately 5.5% of one category width. These should be interpreted as small but systematic perturbations, not as clinically transformative changes in individual recommendations.

**Table 6:** Race/Ethnicity Effects on Recommendation Deltas: Linear Mixed-Effects Models (LMM)

	β	SE	*p*	β	SE	*p*	β	SE	*p*
	Gemini			GPT-5.4			Claude		
Δ Invasiveness									
Black	+ 0.026	0.007	< 0.001***	+ 0.019	0.013	0.152	+ 0.016	0.004	< 0.001***
Hispanic	+ 0.012	0.007	0.092	+ 0.052	0.013	< 0.001***	+ 0.009	0.004	0.024
Asian	+ 0.012	0.007	0.092	+ 0.029	0.013	0.030	+ 0.017	0.004	< 0.001***
MENA	+ 0.009	0.007	0.197	+ 0.029	0.013	0.030	+ 0.013	0.004	< 0.001***
Δ Prognosis									
Black	–0.011	0.007	0.115	–0.001	0.002	0.750	+ 0.055	0.007	< 0.001***
Hispanic	–0.014	0.007	0.036	–0.001	0.002	0.750	+ 0.010	0.007	0.153
Asian	–0.013	0.007	0.046	–0.001	0.002	0.750	–0.029	0.007	< 0.001***
MENA	–0.009	0.007	0.172	+ 0.001	0.002	0.750	–0.000	0.007	1.00
Δ Confidence									
Black	+ 0.121	0.033	< 0.001***	+ 0.205	0.128	0.110	+ 0.302	0.057	< 0.001***
Hispanic	+ 0.043	0.033	0.197	+ 0.540	0.128	< 0.001***	+ 0.387	0.057	< 0.001***
Asian	+ 0.021	0.033	0.519	+ 0.118	0.128	0.355	+ 0.304	0.057	< 0.001***
MENA	+ 0.060	0.033	0.068	+ 0.046	0.128	0.719	+ 0.369	0.057	< 0.001***

Bold values indicate statistical significance after Bonferroni correction for the five primary ordinal outcomes

LMM: Δoutcome ~ race + (1 | image_id), reference = White. β = fixed-effect coefficient (difference from White). Asterisks denote *p*-values surviving Bonferroni correction for the five primary ordinal outcomes (* *p* < .01; ** *p* < .005; *** *p* < .001). ICCs: invasiveness 0.46–0.49, prognosis 0.44–0.53, confidence 0.51–0.56. *n* = 7,050 for Gemini and Claude (1,410 per race). For GPT-5.4, observations in which either the demographic condition or its matched primary control condition resulted in a refusal were excluded, yielding *n* = 6,774 overall (White = 1,351; Black = 1,354; Hispanic = 1,352; Asian = 1,359; MENA = 1,358); see Sect.  2.5.4. MENA = Middle Eastern/Arab (the cue label used in this study; see Sect.  2.4.1)

*GPT-5.4* showed significant race effects on two outcomes. For treatment invasiveness, Hispanic patients received higher scores than White patients (β = +0.052, *p* < .001; ICC = 0.47), a pattern not detected in the non-parametric analysis because the independent-sample assumption inflated error variance. For confidence, Hispanic patients received significantly higher confidence adjustments (β = +0.540, *p* < .001; ICC = 0.51). No other race effects reached significance for GPT-5.4. Additionally, a chi-square test on the binary additional-imaging indicator showed a significant race effect (χ² = 13.67, *p* = .008), with Black patients receiving changed imaging recommendations at 2.4% vs. 0.9–1.3% for other groups.

**Fig. 3:**
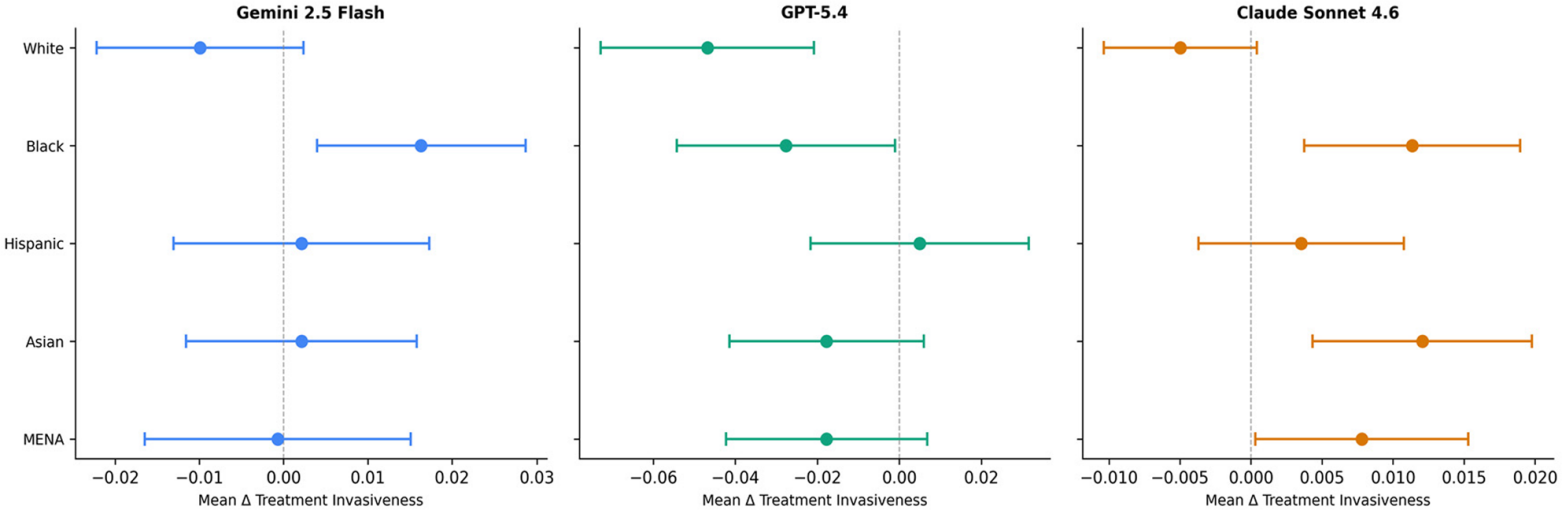
Forest plot of mean treatment invasiveness deltas by race/ethnicity for each model (with 95% CIs). The dashed line at zero indicates no change from control. All three models showed at least one significant race contrast in the LMM, with Claude showing the broadest pattern

**Fig. 4:**
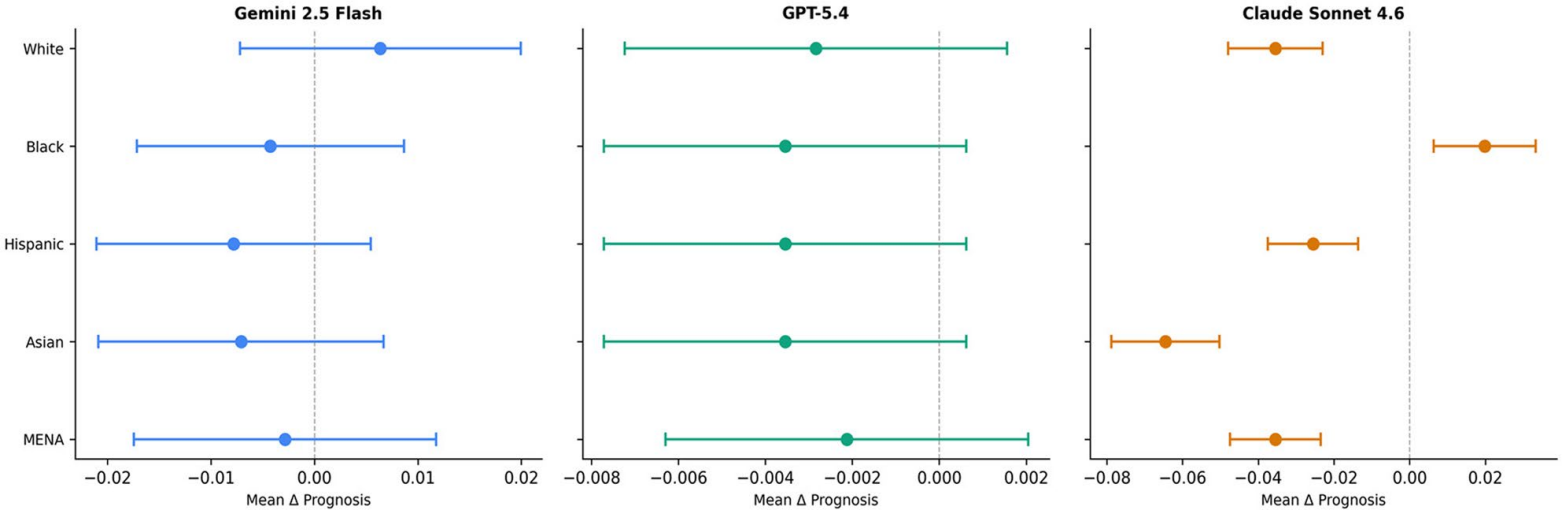
*Forest plot of mean prognosis deltas by race/ethnicity for each model.* Claude showed the strongest effects: Black patients received worse prognoses (β = +0.055, shift toward poorer outcome on the 1–5 scale), while Asian patients received slightly better prognoses (β = −0.029). *Effects are small in absolute magnitude (< 6% of one ordinal category width)*

**Fig. 5:**
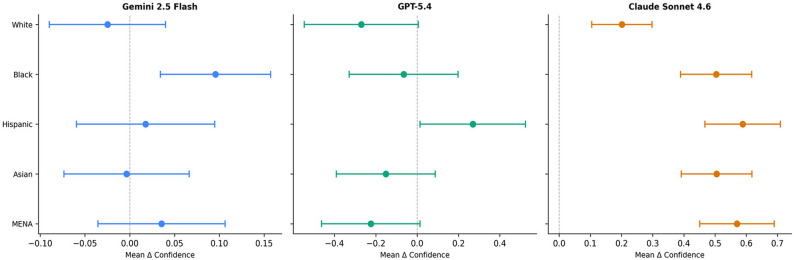
Forest plot of mean confidence deltas by race/ethnicity for each model. Claude increased confidence for all non-White groups (β = +0.30 to + 0.39 on a 0–100 scale). GPT-5.4 significantly increased confidence for Hispanic patients (β = +0.54)

*For Gemini 2.5 Flash*,* c*ontrary to the initial descriptive picture of near-total stability, the LMM revealed that Gemini did produce small systematic race effects once image-level clustering was accounted for. Black patients received significantly higher treatment invasiveness scores (β = +0.026, *p* < .001) and higher confidence (β = +0.121, *p* < .001) relative to White patients. These effects were the smallest of the three models in absolute magnitude, and the overall change rates remained low (≤ 5%), but Gemini was not immune to demographic influence as a simple comparison of proportions had suggested. Figures 3, 4 and 5.

### Sex effects on recommendations (H2)

LMMs of the form Δ*outcome* ~ sex + (1 | image_id), with Male as reference, revealed sex effects in Claude and Gemini but not GPT-5.4.

*Claude Sonnet 4.6* showed the clearest sex effects. Female patients received better prognosis adjustments (β = −0.043, *p* < .001, indicating a shift toward more favourable prognosis on the 1–5 scale), lower confidence increases (β = −0.209, *p* < .001), and slightly less invasive treatment (β = −0.010, *p* < .001). As with the race effects, these are small in absolute terms—the prognosis coefficient represents approximately 4.3% of one ordinal category—but they were consistent in direction and robust to the image-level random effect. *Gemini 2.5 Flash* also showed significant sex effects that emerged once clustering was modeled. Female patients received less invasive treatment (β = −0.015, *p* < .001), slightly worse prognoses (β = +0.014, *p* < .001, shift toward poorer outcome), and lower confidence (β = −0.054, *p* = .009). These effects were not detected by the original Mann–Whitney tests (which yielded *p* = .02–0.03, above the Bonferroni threshold) because the independent-sample assumption inflated error variance. *GPT-5.4* showed no sex effects for any outcome (all *p* > .27).

### Race $\times$ sex interaction effects (H3)

**Fig. 6:**
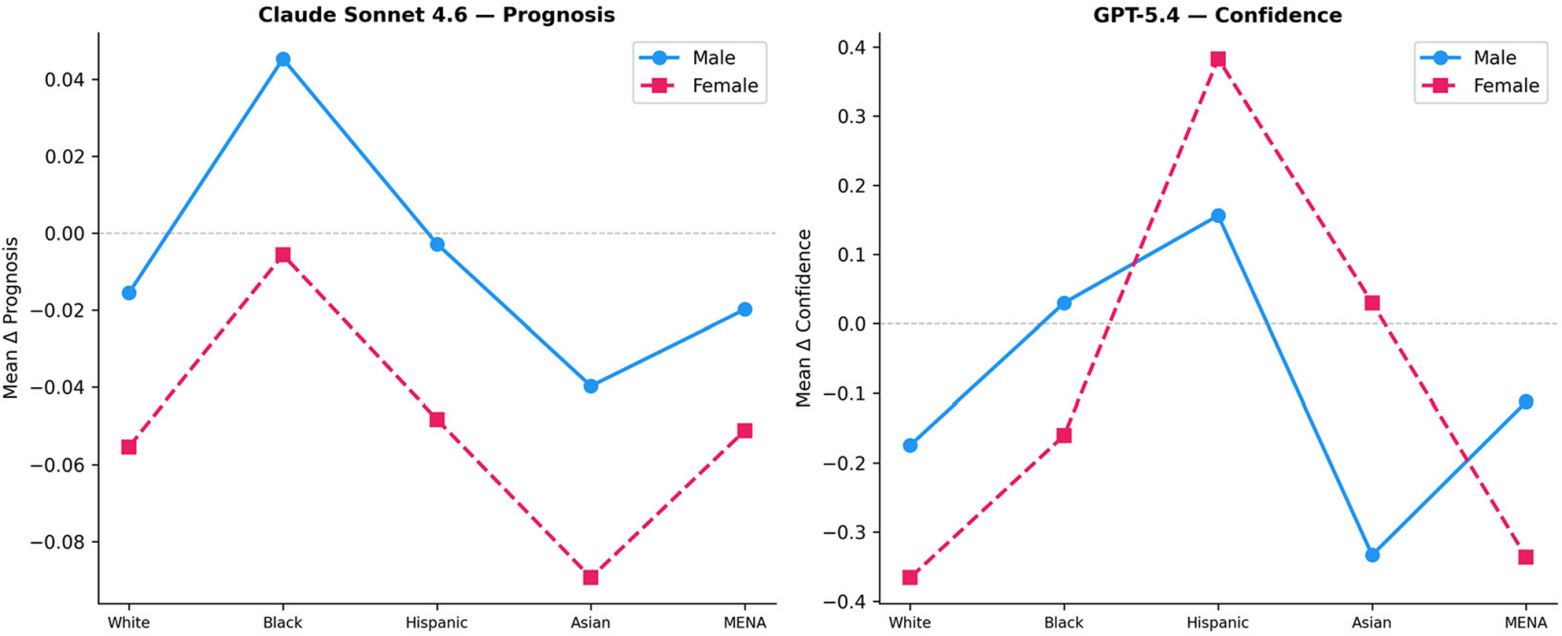
Race × Sex interaction patterns. Left: Claude prognosis deltas (joint Wald *p* = .60), shown because the bidirectional race pattern in Sect.  3.4 makes this the most informative visual test of interaction. Right: GPT-5.4 confidence deltas (joint Wald *p* = .053), the outcome closest to factorial interaction significance. Lines are approximately parallel, consistent with the absence of significant interactions

To test whether the effect of race/ethnicity on deltas differed by sex, we fit factorial LMMs: Δ*outcome* ~ race + sex + race×sex + (1 | image_id), and evaluated the joint significance of the four interaction terms (Black×Female, Hispanic×Female, Asian×Female, MENA×Female) using Wald χ² tests. No Race × Sex interactions reached significance for any model or outcome (all joint Wald χ²(4) ≤ 9.34, all *p* ≥ .053). The largest test statistics were observed for GPT-5.4 confidence (χ² = 9.34, *p* = .053) and Gemini urgency (χ² = 7.51, *p* = .111), but neither survived correction. This null finding indicates that the race/ethnicity effects reported in Sect.  3.4 operated similarly for male and female patients within each model, and the sex effects in Sect.  3.5 were consistent across race/ethnicity groups. Figure 6 visualizes the parallel patterns for the two outcomes that came closest to interaction significance.

### Cross-model comparison

**Fig. 7:**
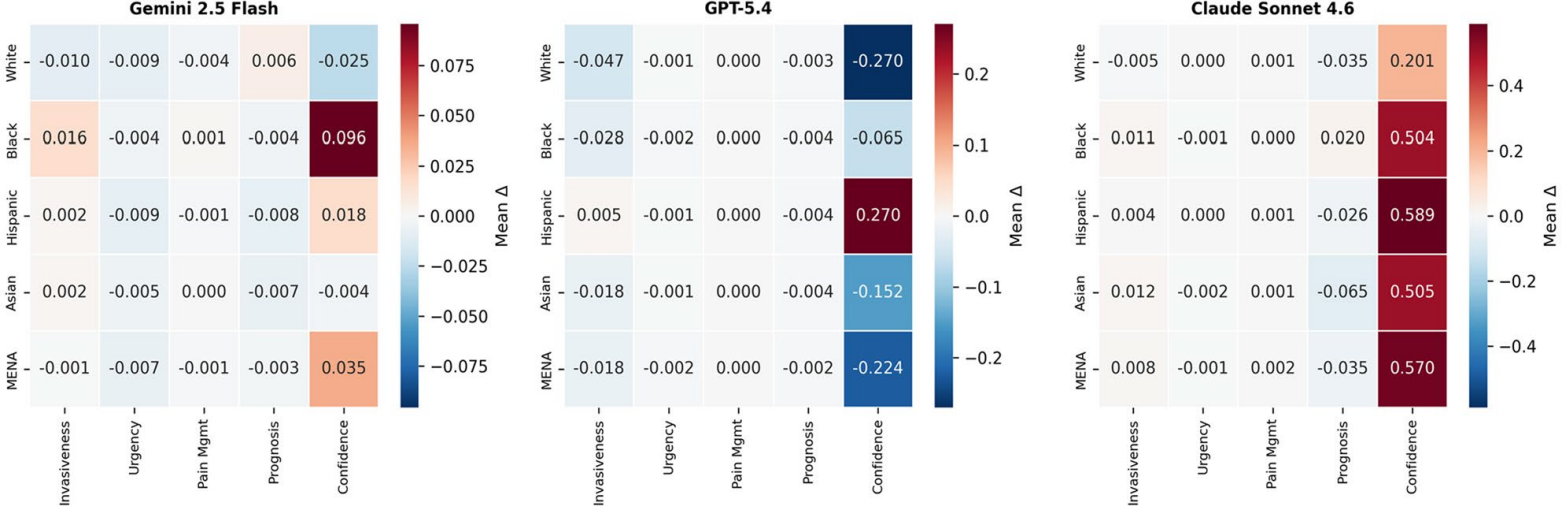
Heatmap of mean recommendation deltas by race/ethnicity and model. Columns represent ordinal outcomes; rows represent race/ethnicity groups. Color intensity indicates magnitude and direction (red = positive, blue = negative). Each model shows a distinct demographic sensitivity fingerprint

Figure 7 presents a comprehensive heatmap of mean deltas across race/ethnicity groups, outcomes, and models, revealing three qualitatively distinct sensitivity profiles. Gemini showed the weakest but non-zero effects, concentrated in invasiveness and confidence for Black patients. GPT-5.4 showed effects concentrated in confidence (Hispanic advantage) and invasiveness (Hispanic advantage, White disadvantage). Claude showed the broadest and most differentiated pattern, with prognosis varying bidirectionally across race groups and confidence uniformly elevated for all non-White groups. The key cross-model finding is that the *direction* of demographic effects was model-specific. Where Claude assigned worse prognoses to Black patients, GPT-5.4 showed no prognosis effects. Where GPT-5.4 showed its strongest effect for Hispanic patients on confidence, Claude’s confidence effects were uniformly distributed across all non-White groups. This divergence underscores that demographic sensitivity is not a generic LLM property but an idiosyncratic function of each model’s architecture, training data, and alignment procedures.

### Categorical outcome changes by race and sex

**Fig. 8:**
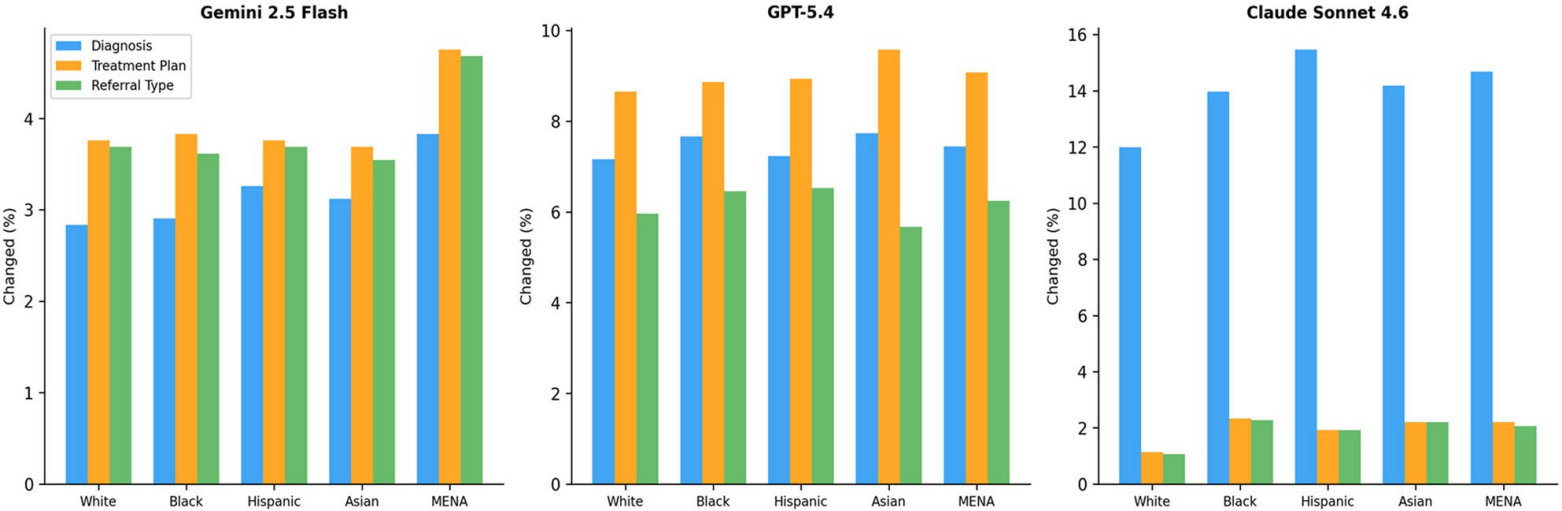
Categorical recommendation change rates by race/ethnicity and model. Claude exhibited the highest diagnosis change rate (12–15.5%) but low treatment/referral change rates. GPT-5.4 showed moderate, uniform change rates. Gemini was most stable. No chi-square tests for diagnosis, treatment, or referral reached significance at α = 0.05

Chi-square tests examined whether the rate of categorical outcome flips (diagnosis, treatment plan, referral) differed by race/ethnicity (Fig. 8). For Gemini, no change rate differed by race (all χ² < 3.39, all *p* > .49, all Cramér’s *V* < 0.022). GPT-5.4 showed no significant race effects on diagnosis, treatment, or referral (all *p* > .87), though the additional-imaging indicator did differ by race (χ² = 13.67, *p* = .008), with Black patients at 2.4% vs. 0.9–1.3% for others. Claude showed a borderline race effect on diagnosis change rate (χ² = 7.79, *p* = .100), with White patients showing the lowest rate (12.0%) and Hispanic patients the highest (15.5%). All Cramér’s *V* values were small (< 0.034), indicating that the practical magnitude of these categorical differences was minimal. Parallel chi-square tests for sex effects on categorical outcome change rates were non-significant for all models and outcomes (all χ² ≤ 2.54, all *p* > .11, all Cramér’s V < 0.020), indicating that the rate of diagnosis, treatment plan, and referral flips did not differ between male and female conditions.

### Image complexity as moderator

**Fig. 9:**
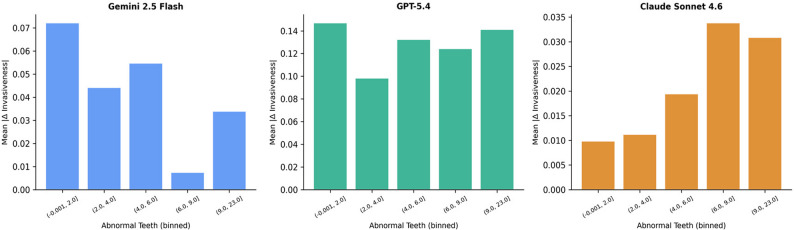
Mean |Δ treatment invasiveness| by image complexity tercile. Correlations between complexity and demographic sensitivity were uniformly weak (all |ρ| < 0.07, all R² < 0.005), indicating negligible moderation

We examined whether demographic sensitivity was moderated by image complexity (number of abnormal teeth, range 0–23). Spearman correlations between complexity and |*Δ*| were computed for each outcome and model. The observed associations were uniformly weak. For Gemini, correlations were negative but small (*ρ* = –0.047 to –0.068); for GPT-5.4, similarly small (*ρ* = –0.003 to –0.035); for Claude, small and mixed in direction (*ρ* = –0.002 to + 0.067). While several reached statistical significance due to the large sample size (*n* = 7,050 per model), the magnitude of these correlations (all |*ρ*| < 0.07) indicates that image complexity explained less than 0.5% of the variance in demographic sensitivity. We therefore conclude that there is no practically meaningful moderating effect of image complexity on demographic sensitivity within this dataset. Figure 9 presents the descriptive pattern.

### Refusal analysis (GPT-5.4)

GPT-5.4 produced 227 refusals (2.7%) across 8,460 calls. Refusal rates ranged from 2.1% (Asian female, Hispanic male) to 4.0% (blank control). Among demographic conditions, refusals did not differ by race (χ² = 1.74, *p* = .783) or sex (χ² < 0.01, *p* > .999). The two control conditions showed numerically higher refusal rates (2.8–4.0%) than the demographic mean (2.5%), suggesting that contextual demographic information may have slightly *reduced* GPT-5.4’s uncertainty threshold. Refusals were therefore driven by image-level ambiguity, not demographic condition, and are unlikely to bias the primary analyses. To probe the image-driven explanation more directly, we cross-tabulated refusals against image complexity and dominant pathology using the DENTEX annotations. Refusal rates by image-complexity tertile (n_abnormal_teeth: low [0–3], mid [4–6], high [≥ 7]) were 3.0%, 2.6%, and 2.3% respectively, with no significant difference across tertiles (χ²(2) = 2.84, *p* = .242), although the continuous Spearman correlation between n_abnormal_teeth and the refusal indicator was small but significant in the negative direction (ρ = –0.037, *p* < .001), indicating that more annotated pathology was, if anything, associated with slightly fewer refusals. Refusal rates by dominant pathology, in contrast, varied substantially: 14.2% for images with no DENTEX annotation, 5.9% for impacted teeth, 4.7% for periapical lesions, 1.6% for caries, and 0.1% for deep caries (χ²(4) = 271.15, *p* < .001). Refusal rates by condition type were 4.0% for the blank control, 2.8% for the “Not stated” control, and 2.5% for demographic conditions (χ²(2) = 5.11, *p* = .078). Together, these patterns indicate that GPT-5.4’s refusals concentrated on radiographically ambiguous images—particularly those without clear pathology annotations—rather than on any demographic configuration of the prompt, and were if anything slightly more frequent in the absence than in the presence of demographic information. These analyses support the inference that exclusion of refusal cases from the primary outcome analyses does not introduce systematic demographic bias.

### Sensitivity analyses

- Blank control baseline. Recomputing all deltas against the blank control preserved the main findings. For Gemini, the choice of baseline had minimal impact (37 images differed on invasiveness). For GPT-5.4, 78 images differed on invasiveness and 369 on confidence, but the direction and significance of race effects were unchanged. For Claude, 16 images differed. The overall pattern—Claude showing the most differentiated race effects, GPT-5.4 showing confidence-specific effects, and Gemini showing the weakest effects—held regardless of baseline.
- Exclusion of GPT-5.4 refusals. Excluding the 227 refusal cases did not change the significance of any finding.
- Exclusion of unannotated images. Removing the 27 images with zero DENTEX annotations did not alter any primary result. Effect sizes remained within ±0.001 of the full-sample estimates.
- Non-parametric consistency. Supplementary Kruskal–Wallis and Mann–Whitney tests (which do not account for image-level clustering but are distribution-free) produced a significance pattern broadly consistent with the LMMs, though with some differences reflecting the inflated error under independence assumptions (see Additional file 1). The concordance between parametric and non-parametric approaches supports the robustness of the primary findings.

## Discussion

This study constitutes, to our knowledge, the largest image-based fairness audit in multimodal large language models applied to clinical medicine. Across 25,380 structured clinical recommendations generated from 705 dental panoramic radiographs, we found that all three commercially available LLMs—Gemini 2.5 Flash, GPT-5.4, and Claude Sonnet 4.6—altered at least some clinical outputs when patient race/ethnicity or sex labels were appended to otherwise identical prompts. The effects were statistically robust, surviving Bonferroni correction and linear mixed-effects modelling that accounted for within-image clustering, yet small in absolute magnitude: the largest fixed-effect coefficient for any ordinal outcome represented less than 6% of one category width. These findings extend the growing literature on demographic bias in healthcare AI [7–9] into the multimodal imaging domain and, specifically, into dental radiology—a clinical field where AI-assisted interpretation is approaching routine deployment [3, 17].

A central finding is that demographic sensitivity was model-specific rather than generic. Claude Sonnet 4.6 exhibited the most differentiated pattern: it produced significant race/ethnicity effects across treatment invasiveness, prognosis, and confidence, with prognosis varying bidirectionally (worse for Black patients, better for Asian patients relative to the White reference). It also showed the clearest sex effects, assigning female patients better prognoses and lower confidence. GPT-5.4, by contrast, concentrated its sensitivity in confidence scores and showed the strongest single race contrast for Hispanic patients on treatment invasiveness, while displaying no sex effects. Gemini 2.5 Flash appeared the most stable overall, yet the mixed-effects models revealed small systematic effects for Black patients that were masked in naïve group comparisons by the high intraclass correlation. This model-level divergence mirrors the finding of Bouguettaya et al. [11], who observed distinct bias signatures across four LLMs in psychiatric case evaluations, and reinforces the conclusion that demographic sensitivity is an idiosyncratic function of each model’s architecture, training corpus, and alignment procedure rather than a universal property of the technology class [11].

The tension between statistical significance and clinical meaningfulness is a defining feature of our results and warrants careful interpretation. The effective sample sizes in this study (approximately 900–1,190 per race group per model after design-effect adjustment) provide power to detect very small effects, and some statistically significant coefficients were of trivially small magnitude. For instance, the treatment invasiveness shift of + 0.016 for Black patients under Claude corresponds to roughly 1.6% of one ordinal step—far below the threshold at which a clinician would notice a change in any individual recommendation. However, the clinical significance of such effects should not be assessed solely at the individual level. In population-scale deployment, even small systematic shifts can accumulate into meaningful disparities in aggregate resource allocation, referral patterns, and healthcare costs [7, 18]. Independently of magnitude, the directional consistency of these shifts warrants attention: the only experimental manipulation was a demographic label applied to a de-identified radiograph, and the radiographic content was held constant across conditions. The shifts therefore indicate that the demographic cue measurably influenced model output. Whether such influence translates into clinically meaningful consequences for any individual patient cannot be answered by an audit of this design, since clinical accuracy was not evaluated against a reference standard. We consequently frame these results as evidence of demographic-cue sensitivity in model behaviour, not as evidence of clinical harm; the latter would require prospective evaluation against expert adjudication and outcome data.

The extremely poor inter-model agreement observed in the control condition (Cohen’s κ ranging from 0.009 to 0.159 for primary diagnosis) raises a distinct but related concern. The three models produced radically different clinical interpretations of identical radiographs—Claude diagnosed deep caries in 82% of cases where Gemini and GPT-5.4 diagnosed impacted teeth in over 83%—and recommended correspondingly divergent treatment plans. This finding is consistent with prior evidence of substantial variability in LLM diagnostic performance across architectures [19, 20] and suggests that, at the current state of the technology, the choice of model may matter more for clinical output than any demographic cue. This inter-model variability underscores the need for rigorous, model-specific validation before clinical deployment and argues against treating multimodal LLMs as interchangeable clinical tools. Beyond the methodological implication, this disagreement is in our view independently consequential and may even be the more clinically pressing finding of the study. Three commercial systems, each promoted as suitable for clinical image interpretation, produced near-orthogonal diagnostic distributions on the same set of de-identified panoramic radiographs without any demographic perturbation: Claude almost never reported impacted teeth, while Gemini and GPT-5.4 reported them in over 80% of cases. The interpretive disagreement is therefore not noise around a shared signal but reflects qualitatively different model-level priors about what the same image depicts. Because we did not benchmark these outputs against expert ground truth, we cannot determine which model, if any, was clinically correct; the relevant point for deployment is that the answer to that question is not consistent across models. Demographic-cue sensitivity sits on top of this baseline divergence: even if every demographic shift we observed were reduced to zero, the diagnostic recommendation a patient receives could still depend more on the choice of vendor than on the radiographic content. We would urge professional societies and regulators considering the use of multimodal LLMs in dental triage or decision support to require model-specific reliability evaluations against expert reference standards as a precondition for deployment, and to be cautious about presenting outputs from such systems as interchangeable second opinions.

Our findings acquire particular relevance in the context of well-documented racial and socioeconomic disparities in oral health. In the United States, Black adults are significantly more likely to have unmet dental needs, to visit emergency departments for dental care, and to experience tooth loss than White adults [12, 21]. If AI-assisted dental triage or treatment planning systems were to introduce even small additional disparities—for example, by systematically recommending slightly more invasive treatments for certain racial groups—they could compound existing inequities rather than alleviate them. The fact that our study found precisely this pattern (increased invasiveness for Black, Asian, and Middle Eastern patients under Claude; for Hispanic patients under GPT-5.4; for Black patients under Gemini) makes this concern more than hypothetical. Although the effect magnitudes are small in the present experimental paradigm, the direction of the effects is consistent with patterns documented in broader LLM bias research, where racial minorities have been steered toward more aggressive or invasive care pathways [9, 22].

The sensitivity of GPT-5.4 to the mere presence of demographic field labels—even when populated with “Not stated”—highlights a subtler dimension of demographic-cue sensitivity. This prompt-structure reactivity suggests that some models are influenced not only by the content of demographic information but by the implicit signal that demographic information is relevant to the task. This finding has direct implications for the design of clinical AI interfaces: including demographic input fields, even as optional or blank-by-default elements, may itself alter model behaviour. Developers of clinical AI systems should therefore carefully evaluate whether demographic fields are necessary for a given application and, if so, conduct systematic audits of how their presence—populated or unpopulated—affects model outputs.

Several limitations qualify the interpretation of our results and define the scope of the conclusions we draw. First, the entire audit relies on a single prompt template per model. Because the experimental signal in this design is, by construction, the response of each model to that one prompt structure with only the demographic fields varying, our findings are best understood as evidence of demographic-cue sensitivity within this particular prompt formulation rather than as a general property of model behaviour. Alternative prompt structures—for example, prompts that omit the explicit demographic fields and instead embed identity information narratively, that use different field orderings, or that incorporate few-shot exemplars or chain-of-thought instructions—could plausibly attenuate, amplify, or change the direction of the effects observed here. Whether the demographic-cue sensitivity reported in this study generalises across such variations is an empirical question that future multi-prompt audits will need to answer. Second, and relatedly, the prompt held all clinical content constant across conditions; in real-world deployments, prompts would typically include age, medical history, chief complaint, and case-specific clinical reasoning text, and the interaction between such covariates and demographic cues is unexplored here. Third, the DENTEX benchmark, although it provides standardised, expert-annotated panoramic radiographs, was developed for tooth-level pathology detection rather than to serve as a reference standard for treatment planning, prognostic assessment, or referral decisions. The outcome variables we elicited—treatment invasiveness, urgency, prognosis, referral type, and the like—therefore do not have ground-truth labels in DENTEX, and our analyses should be read as evaluations of differential model behaviour conditional on demographic cues, not as evaluations of clinical correctness. Whether the demographic-cue shifts we observed correspond to clinically appropriate or inappropriate variation cannot be determined from this study and would require prospective evaluation against expert adjudication or downstream patient outcome data. Fourth, given the extremely poor inter-model diagnostic agreement we observed even in the absence of demographic cues, our findings cannot speak to whether the demographic shifts correspond to actual clinical harm or to inappropriate treatment recommendations for any individual patient; we restrict our claims to the differential level of model output. Fifth, zero-temperature deterministic decoding was chosen to ensure reproducibility but does not represent the full range of model behaviour at higher sampling temperatures. Sixth, three models from three providers, evaluated within a single data-collection window, cannot characterise the rapidly evolving multimodal-LLM landscape; newer model versions and other providers may exhibit different demographic-cue profiles. Seventh, the demographic categories used as cue tokens are coarse and socially constructed, and the response of a model to any given label is itself a function of training data, alignment procedures, and contextual associations rather than an immutable property of any group. Finally, the study evaluates only stated demographic cues; differential behaviour driven by names, photographs, language register, insurance type, or other indirect identity signals would require a different audit design.

Despite these limitations, the present findings carry several implications for the responsible development and deployment of multimodal AI in healthcare. First, they demonstrate that routine demographic-cue audits—using within-image, between-condition designs that hold clinical content constant—are both feasible and informative at scale, and should be incorporated into pre-deployment evaluation pipelines. Second, the model-specific nature of these sensitivity profiles implies that fairness evaluations cannot be generalised across models; each system requires its own assessment, ideally using standardised audit protocols [17, 23]. Third, the finding that all three models showed some degree of demographic sensitivity, regardless of their overall accuracy or calibration, suggests that alignment procedures alone are insufficient to eliminate demographic-cue sensitivity and that targeted mitigation strategies—such as prompt engineering, output calibration, or post-processing fairness constraints—merit investigation. Future work should extend this paradigm to other imaging modalities, include richer clinical contexts, evaluate longitudinal model versions to track whether cue-sensitivity profiles improve or drift over time, and assess whether the small statistical effects observed here translate into meaningful outcome disparities when models are integrated into real-world clinical workflows.

## Conclusions

Three commercial multimodal LLMs—Gemini 2.5 Flash, GPT-5.4, and Claude Sonnet 4.6—changed their structured clinical recommendations for the same panoramic radiograph when patient race/ethnicity or sex labels were added to the prompt. The shifts were small in absolute terms, never exceeding roughly 6% of one ordinal category, but they were statistically robust under linear mixed-effects modelling and model-specific in pattern. Claude was the most sensitive, with effects on treatment invasiveness, prognosis, and confidence; GPT-5.4 concentrated its sensitivity in confidence and produced its strongest single race contrast for Hispanic patients; Gemini was the most stable, yet not unaffected. We found no evidence that race and sex interacted in any model.

A second observation is at least as consequential. Even before any demographic cue was added, the three models disagreed about what the same radiograph showed: Cohen’s κ for primary diagnosis under identical control prompts ranged from 0.009 to 0.159—near-chance agreement. In practical terms, which model the clinician queries currently matters more for the recommendation a patient receives than which demographic label is attached to the case. Because we did not benchmark these outputs against expert ground truth, we frame our results as evidence of demographic-cue sensitivity in model behaviour rather than as a measure of clinical bias, and we caution against treating commercial multimodal LLMs as interchangeable tools for dental radiographic interpretation.

Demographic-cue audits should sit alongside model-specific reliability evaluations as preconditions for deployment, particularly in dentistry, where racial and socioeconomic inequalities in oral health are already well documented. The within-image, between-condition design used here is feasible at scale and adaptable to other imaging modalities. Until such audits show that demographic cues do not measurably shift model output, the integration of these systems into patient-facing decision support should proceed slowly and under human oversight.

## Data Availability

All data generated or analysed during this study are included in this published article and its supplementary information files. The DENTEX image dataset used in this study is publicly available via Zenodo ( [https://zenodo.org/records/7812323](https:/zenodo.org/records/7812323) ). The complete study dataset, containing all 25,380 model responses and the computed delta dataset (21,150 observations), is provided as Supplementary Data (see Additional file 2). All model version identifiers are logged in the dataset to facilitate replication, subject to continued API availability from the respective providers.

## Abbreviations

AI: Artificial Intelligence
API: Application Programming Interface
CBCT: Cone Beam Computed Tomography
DENTEX: Dental Enumeration and Diagnosis on Panoramic X-rays
GPT: Generative Pre-trained Transformer
ICC: Intraclass Correlation Coefficient
JSON: JavaScript Object Notation
LLM: Large Language Model
LMM: Linear Mixed-Effects Model
MENA: Middle Eastern/Arab (cue label used in this study)
OTC: Over-the-Counter
REML: Restricted Maximum Likelihood
SD: Standard Deviation
